# Detection of imprinting and heterogeneous maternal effects on high blood pressure using Framingham Heart Study data

**DOI:** 10.1186/1753-6561-3-s7-s125

**Published:** 2009-12-15

**Authors:** Jingyuan Yang, Shili Lin

**Affiliations:** 1 Department of Statistics, The Ohio State University, 1958 Neil Avenue, Cockins Hall, Room 404, Columbus, Ohio 43210-1247, USA

## Abstract

Both imprinting and maternal effects could lead to parent-of-origin patterns in complex traits of human disorders. Statistical methods that differentiate these two effects and identify them simultaneously by using family-based data from retrospective studies are available. The usual data structures include case-parents triads and nuclear families with multiple affected siblings. We develop a likelihood-based method to detect imprinting and maternal effects simultaneously using data from prospective studies. The proposed method utilizes both affected and unaffected siblings in nuclear families by modeling familial genotypes and offspring's disease status jointly. Maternal effect is usually modeled as a fixed effect under the assumption that maternal variant allele(s) has (have) identical effect on any offspring. However, recent studies report that different people may carry different amounts of substances encoded by the mother's variant allele(s) (called maternal microchimerism), which could result in heterogeneity of maternal effects. The proposed method incorporates the heterogeneity of maternal effects by adding a random component to the logit of the penetrance. Our method was applied to the Framingham Heart Study data in two steps to detect single-nucleotide polymorphisms (SNPs) that may be associated with high blood pressure. In the first step, SNPs that affect susceptibility of high blood pressure through minor allele, genomic imprinting, or maternal effects were identified by using the proposed model without the random effect component. In the second step, we fitted the mixed effect model to the identified SNPs that have significant maternal effect to detect heterogeneity of the maternal effects.

## Background

The phenomenon that a trait follows a maternal or paternal lineage, instead of following the mendelian mode of inheritance, is referred to as the parent-of-origin pattern. Genomic imprinting and maternal effect could give rise to similar parent-of-origin patterns [1]. Hence, models, which are designed to identify genomic imprinting by detecting the parent-of-origin pattern, may report false positives that are actually due to maternal effect. A log-linear likelihood-ratio test (LL-LRT) [2,3] was developed to detect imprinting and maternal effects simultaneously by using case-parents triads. This approach was then extended to the parent-of-origin likelihood ratio test (PO-LRT) to detect imprinting and maternal effects at a marker that is in linkage disequilibrium with a candidate gene [4].

A case-parents triad could have 15 possible familial genotype combinations [2-4]. LL-LRT and PO-LRT model the counts of the 15 categories by using log-linear and logistic regression, respectively. It has been shown that these methods are robust for detecting imprinting effect even in the presence of maternal effect. However, when multiple affected siblings are genotyped (e.g., the North American Rheumatoid Arthritis Consortium data set [5]), trimming nuclear families to case-parents triads does not make the most of the data and thus limits the power. In contrast, the maternal-fetal genotype incompatibility (MFG) test [6] could model nuclear families with multiple affected siblings. Compared with the generalized linear models LL-LRT and PO-LRT, it does require more effort in formulating the likelihood to implement the MFG test. The MFG test was developed to detect maternal effect and an interaction effect between the mother carrying one copy of the disease susceptibility allele and the child carrying no copy under the assumption of no imprinting.

Unaffected siblings are also genotyped in many genetic studies. Although unaffected siblings have been incorporated to infer the missing parental genotypes in LL-LRT and PO-LRT [7], they do not directly contribute to model the relative risks due to genomic imprinting and maternal effect. LL-LRT, PO-LRT, and MFG tests only model affected sibling(s) because they all use the data from retrospective studies. Sampling from a retrospective study is biased because only families with affected child(ren) are recruited.

On the other hand, a prospective study like the Framingham Heart Study (FHS) does not specifically recruit subjects with a certain disease, thus the originally recruited cohort could be considered as a random sample of the general population of healthy people and patients with any disease. Therefore, a test using data from a prospective study could utilize both affected and unaffected siblings by modeling their genotypes and disease status jointly. Nevertheless, the disease of interest should not be a rare one; otherwise, the number of patients in the cohort would be too small to be sufficiently informative.

In LL-LRT, PO-LRT, and MFG tests, maternal effect is modeled as a fixed effect because it is assumed that maternal variant allele(s) has (have) identical effect on any offspring. This assumption might be invalid if we consider the cause of maternal effect more carefully. Maternal effect refers to the phenomenon that the genotype of a mother is expressed in the phenotype of her offspring, which is usually attributed to maternally produced molecules, such as mRNA that are deposited in the egg cell, and mRNA or antigens that are passed to the offspring during pregnancy. The latter case could arise from a biological process called microchimerism. Microchimerism means that two genetically distinct cells, one being at a low concentration, are present in the same individual. Microchimerism may be due to transfer of cells between mother and fetus or between two twins. Other sources of microchimerism include blood transfusions and organ transplants. Cells transferred from the mother to the fetus are referred to as maternal microchimerism (MMc). Non-inherited maternal antigen coding alleles (NIMA) [5,6] within MMc would be expressed in the offspring and increase his or her susceptibility to a certain disease. It has been found by quantitative real-time polymerase chain reaction that different individuals may have different amounts of MMc. Therefore, it is likely that the maternal effects imposed by different mothers are actually heterogeneous rather than being homogenous as assumed in the previous studies.

In this study, we will discuss a prospective likelihood formulation that will take multiple affected and unaffected siblings into consideration. Further, we will treat maternal effects as random to model heterogeneity. This method will then be applied to the high blood pressure (HBP) trait in the FHS.

## Methods

### Definition of HBP trait in FHS

HBP in an adult is defined as a blood pressure greater than or equal to 140 mm Hg systolic pressure or greater than or equal to 90 mm Hg diastolic pressure. High blood pressure directly increases the risk of coronary heart disease and stroke, especially when it is present with other risk factors. In the FHS, systolic pressure and diastolic pressure of the Original Cohort (the first generation) and their Offspring Cohort (the second generation) were measured at four exams, and were measured once in the Generation 3. Based on the highest measurements among all available ones, there were 1,036 individuals having high blood pressure and 1,724 individuals having normal blood pressure in the Offspring Cohort. In Generation 3, there were 379 individuals having high blood pressure and 3,618 individuals having normal blood pressure. Our analysis was based on these phenotypic data and the genotypes of nuclear families. Families with missing parental genotype(s) were omitted from our analysis because they are not informative for imprinting and maternal effects if both parents' genotypes are missing.

Moreover, only one nuclear family was selected at random to be included in the analysis from each three-generation pedigree. This selection process led to approximately 300 nuclear families (with the number of children in each family ranging from 1-8) for each single-nucleotide polymorphism (SNP).

### Prospective likelihood and random effect modeling

Suppose there are *N *nuclear families. The *i*^th ^family has two parents and *n*_*i *_children, *i *= 1, 2, ..., *N. *We model the genotypes of all family members and disease statuses of all children jointly. Let *M*_*i *_and *F*_*i *_denote the genotypes of mother and father in the *i*^th ^family.

 denotes the genotypes of *n*_*i *_children in the *i*^th ^family. *M*_*i*_, *F*_*i*_, and  could take a value of 0, 1, or 2, indicating the number of the minor allele(s) at a SNP carried by the corresponding person, *j *= 1, 2, ..., *n*.  denotes the sibling's disease status, with  indicating that the *j*^th ^child in the *i*^th ^family being affected, and 0 indicating otherwise.

The likelihood for the *i*^th ^family could be decomposed as

We use the logit model to relate the penetrance to the imprinting effect (part of the *β *parameters) and the maternal effect (the *γ *parameters):

where  is a vector with the covariates related to the *j*^th ^offspring in the *i*^th ^family coded in one row:  and this single copy is inherited from the mother), *I*(*M*_*i *_= 1), *I*(*M*_*i *_= 2)). The parameter vector , where *β*_0 _is the logit of phenocopy rate; *β*_1 _measures the effect of the single minor allele inherited from the father; *β*_1 _+ *β*_*im *_measures the effect of the single minor allele inherited from the mother; *β*_*im *_measures the imprinting effect, with *β*_*im *_< 0 indicating maternal imprinting and *β*_*im *_> 0 indicating paternal imprinting; *β*_2 _measures the effect of two copies of the minor allele; *γ*_1 _and *γ*_2 _measure the maternal effect when the mother carries one or two copies of the minor allele, respectively. Because we assume that different mothers may impose heterogeneous maternal effects, *ε*_*i *_measures the deviation of the effect size from the mean; it also introduces correlation among the siblings within the same family. Specifically, *ε*_*i *_is assumed to follow *N*(0, *σ*^2^).

The parameters *β*_0_, *β*_1_, *β*_2_, *β*_*im*_, *γ*_1_, *γ*_2_, and *σ*^2 ^are estimated by maximizing the likelihood using the procedure NLMIXED in SAS.

### Selection of SNPs

SNPs that may have a minor allele effect, imprinting effect, or maternal effect are selected in the first step and the heterogeneity of maternal effects is detected in the second step. In the first step, we fit the parsimonious fixed-effect model without the random component *ε*_*i *_and use the minimum *p*-value (among the tests for *β*_1 _= 0, *β*_2 _= 0, *β*_*im *_= 0, *γ*_1 _= 0, and *γ*_2 _= 0) being ≤ 0.00005 as the criterion to choose the SNPs. We then screen the selected SNPs by checking specific parameter constraints. The assumption that carrying minor alleles would increase the disease risk implies that *β*_1 _≥ 0 and *β*_1 _+ *β*_*im *_≥ 0. Furthermore, two copies of the minor allele should have an effect at least as large as a single copy, and thus *β*_2 _≥ max(*β*_1_, *β*_1 _+ *β*_*im*_). Estimates of *β*_1 _and *β*_2 _being significantly less than zero indicates that labels of minor and major alleles should be reversed. SAS does not allow complex constraints such as *β*_1 _+ *β*_*im *_≥ 0 and *β*_2 _≥ max(*β*_1_, *β*_1 _+ *β*_*im*_) in its NLMIXED procedure, and as such we fit a "constraint model" and an "additive model" to check the intended constraints. In the "constraint model", we reparametrize *β*_2 _- *β*_1 _=  and impose *β*_1 _≥ 0 and  ≥ 0 to ensure that *β*_1 _≥ 0 and *β*_2 _≥ 0. In the "additive model", we assume an additive effect of the two copies of the minor allele, i.e., *β*_2 _= *β*_1 _+ (*β*_1 _+ *β*_*im*_), to ensure that *β*_2 _≥ max(*β*_1_, *β*_1 _+ *β*_*im*_). In the second step, we fit the mixed effect model to the selected SNPs with significant maternal effect and use the *p*-value of testing *σ *= 0 being less than 0.05 and reduced Akaike information criterion (AIC) value as the criterion for identifying heterogeneity of maternal effects.

## Results

We scanned 230 k SNPs on chromosomes 1 to 6 and detected nine SNPs that may be associated with high blood pressure through minor allele, imprinting, or maternal effect. These nine SNPs are shown in Table 1. The *p*-values for testing *β*_1 _= 0 or *β*_2 _= 0 being less than 0.05 are shown in boldface, which signifies potential minor allele effects. For SNPs that have significant minor allele effect, we further looked at the *p*-value for testing *β*_*im *_= 0 and inferred maternal or paternal imprinting effect from the sign of . If the *p*-value for testing *γ*_1 _= 0 or *γ*_2 _= 0 is less than 0.05, we further tested for heterogeneity of the maternal effects in step two. Results from the second step are shown in Table 2. There are two rows for each SNPs detected to have potential significant maternal effect. The first row shows the estimates, *p*-values, and AIC of the fixed effect model, while the second row shows those of the mixed effect model. If the *p*-value for testing *σ *= 0 is less than 0.05 and the AIC is reduced by fitting the mixed effect model, we concluded that the maternal effect is heterogeneous. Five SNPs were detected to have various degrees of maternal effects, of which four appears to be heterogeneous among the families (Table 2).

**Table 1:** Parameter estimates and *p*-values of the fixed effect model in the first step

Location	SNP	Association with BP	**and *p*-value**		**and *p*-value**		**and *p*-value**		**and *p*-value**		**and *p*-value**	
Chr2 p22.3	rs1979148	Rat	0.406	0.3365	-3.579	0.2899	-1.445	0.0200	1.648	**0.0000**	1.769	0.0517
Chr1 q41	rs17476063	Rat	0.661	0.0883	-0.650	0.3358	-2.201	0.0015	1.644	**0.0000**	0.441	0.7048
Chr4 q26	rs1459543	Human	1.957	**0.0000**^a^	1.296	**0.0146**	-1.586	**0.0109**	0.346	0.3935	0.100	0.8677
Chr1 p36.22	rs6688233	Human, Rat	1.573	**0.0000**	0.234	0.7368	-1.609	**0.0112**	0.377	0.3854	0.747	0.3410
Chr6 q24.1	rs4386830	Human	1.046	**0.0178**	-1.018	0.0809	-2.414	**0.0003**	1.741	**0.0001**	3.031	**0.0000**
Chr3 p14.1	rs13076104	Rat	1.323	**0.0000**	-0.008	0.9910	-1.154	0.0820	0.141	0.7209	0.487	0.5428
Chr2 q24.1	rs10209732	Human, Rat	0.469	0.3301	-1.535	0.1515	-1.089	0.1160	1.632	**0.0000**	1.746	0.0619
Chr3 q26.1	rs2030350	Human, Rat	-0.260	0.5752	-0.902	0.1503	-1.856	0.0202	1.422	**0.0000**	0.726	0.5396
Chr3 p12.1	rs9866277	Rat	1.442	**0.0000**	0.100	0.9367	-0.488	0.5423	-0.565	0.3501	-0.361	0.7332

^a^Bold font under , , , and  indicates *p*-value ≤ 0.05. Bold font under  indicates *p*-value ≤ 0.05 and at least one *p*-value ≤ 0.05 for testing whether *β*_1 _= 0 and *β*_2 _= 0

**Table 2:** Parameter estimates, *p*-values, and AICs of the fixed and mixed effect models in the second step

Location	SNP	**and *p*-value**		**and *p*-value**		**and *p*-value**		**and *p*-value**		**and *p*-value**		**and *p*-value**		AIC
Chr2 p22.3	rs1979148	0.406	0.3365	-3.579	0.2899	-1.445	0.0200	1.648	0.0000	1.769	0.0517	---	---	1548.1
		0.807	0.3500	-2.256	0.5740	-2.469	0.0328	1.886	0.0381	2.273	0.3364	6.771	**0.0000**^a^	1523.6
Chr1 q41	rs17476063	0.661	0.0883	-0.650	0.3358	-2.201	0.0015	1.644	0.0000	0.441	0.7048	---	---	1797.4
		0.630	0.4079	-2.637	0.2000	-4.562	0.0035	2.041	0.0212	2.125	0.5012	6.775	**0.0001**	1782.0
Chr6 q24.1	rs4386830	1.046	0.0178	-1.018	0.0809	-2.414	0.0003	1.741	0.0001	3.031	0.0000	---	---	1847.1
		1.950	0.0936	-4.746	0.0027	-3.739	0.0198	3.017	0.0339	5.217	0.0116	8.388	**0.0000**	1822.6
Chr2 p24.1	rs10209732	0.469	0.3301	-1.535	0.1515	-1.089	0.1160	1.632	0.0000	1.746	0.0619	---	---	1655.1
		0.361	0.5324	-1.589	0.1841	-1.204	0.1482	1.834	0.0005	2.038	0.0902	1.596	**0.0164**	1653.3
Chr3 q26.1	rs2030350	-0.260	0.5752	-0.902	0.1503	-1.856	0.0202	1.422	0.0000	0.726	0.5396	---	---	1704.6
		0.003	0.9957	-1.443	0.1047	-2.709	0.0276	1.840	0.0033	1.439	0.3453	1.925	0.0798	1700.4

^a^Bold font indicates *p*-value ≤ 0.05 for testing whether *σ *= 0

**Figure 1:**
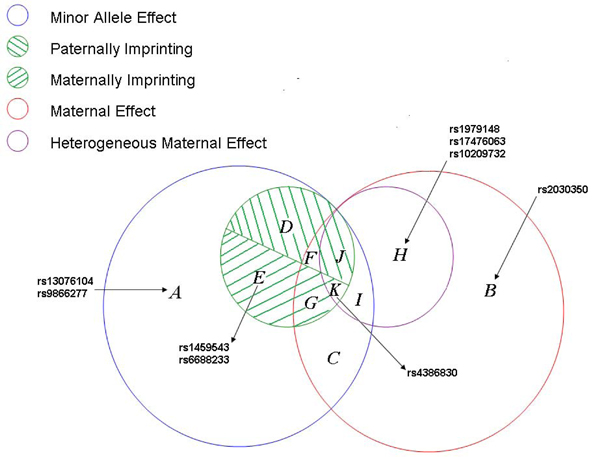
**Venn diagram**.

To summarize and visualize the result, we categorize the nine SNPs into a Venn diagram (Figure 1) according to how the SNPs are associated with high blood pressure. SNPs listed in the intersecting areas have the corresponding effects shown in the legend simultaneously. The locations of the nine detected SNPs in human genome are shown in the first column of Table 1.

## Discussion

In the Genetic Association Studies of Complex Diseases and Disorders section of the Genome Browser, we found that the association between the nine detected SNPs and blood pressure has been established either in human or in rat (see the third column of Table 1). This finding confirms the effectiveness of our method in detecting genetic association. Although the associations of five of these nine SNPs have been reported in human studies, we have found the associations of four additional SNPs in humans, rs1979148, rs17476063, rs13076104, and rs9866277, which were only reported in rat before. Furthermore, there is no previous report of imprinting or maternal effect for any of the nine detected SNPs, whereas our model detected potential imprinting effects for three and maternal effects for five of the SNPs. Finally, we note that, in our current formulation, siblings sharing the same mother have the same maternal effect component, and so it would be interesting to consider hierarchical modelling to further delineate maternal effect heterogeneity among offspring of the same mother. However, random inheritance of the minor allele from mother and father would impose different imprinting component on them, which helps to separate the maternal effect from the imprinting effect. Indeed, we found that our method could detect maternal and imprinting effects simultaneously with reasonable power via simulation.

## List of abbreviations used

AIC: Akaike information criterion; FHS: Framingham Heart Study; HBP: High blood pressure; LL-LRT: Log-linear likelihood ratio test; MFG: maternal-fetal genotype incompatibility; MMc: Maternal microchimerism; NIMA: Non-inherited maternal antigen coding alleles; PO-LRT: Parent-of-origin likelihood-ratio test; SNP: Single-nucleotide polymorphism.

## Competing interests

The authors declare that they have no competing interests.

## Authors' contributions

JY and SL developed the methodology and designed the analysis scheme. JY implemented the analysis and drafted the manuscript. Both authors read and approved the final manuscript.
